# Predators and Resources Influence Phosphorus Transfer along an Invertebrate Food Web through Changes in Prey Behaviour

**DOI:** 10.1371/journal.pone.0065186

**Published:** 2013-06-04

**Authors:** Edoardo Calizza, Loreto Rossi, Maria Letizia Costantini

**Affiliations:** Department of Environmental Biology, ‘Sapienza’ University of Rome, Rome, Italy; University of Western Ontario, Canada

## Abstract

Predators play a fundamental role in prey trophic behaviour, with indirect consequences for species coexistence and ecosystem functioning. Resource quality and availability also influence prey trophic behaviour, with potential effects on predator-prey dynamics. Although many studies have addressed these topics, little attention has been paid to the combined effects of predators and resources on prey species coexistence and nutrient transfer along food chains, especially in detritus-based systems. To determine the influence of predators and resource quality on the movement and P uptake of detritivores, we carried out a field experiment on the River Kelvin (Scotland) using ^32^P to test the hypothesis of reduced prey vagility among resource patches as a strategy to avoid predation. Thirty leaf sacks containing alder leaves and two detritivore prey populations (*Asellus aquaticus* and *Lymnaea peregra*) were placed in cages, half of them with two predator species (*Dendrocoelum lacteum* and *Erpobdella octoculata*) and the other half without predators. Five alder leaf bags, each individually inoculated with a different fungus strain to simulate a patchy habitat, were placed inside each leaf sack. One bag in each sack was labelled with ^32^P, in order to assess the proportion of detritivores using it as food and thus their movement among the five resource patches. Three replicates for each labelled fungus and each predation treatment (i.e. with and without predators) were left on the riverbed for 7 days. The presence of predators had negligible effects on the number of detritivores in the leaf bags, but it did reduce the proportion of ^32^P-labelled detritivores and their P uptake. The most strongly affected species was *A. aquaticus*, whose vagility, trophic overlap with *L. peregra* and P uptake were all reduced. The results confirm the importance of bottom-up and top-down forces acting simultaneously to regulate nutrient transfer along food chains in patchy habitats.

## Introduction

Top-down and bottom-up interactions play a fundamental role in the structure and dynamics of ecological communities [1], [2], [3], with important implications for species coexistence and ecological processes [4], [5], [6], [7]. Prey species can modify their spatial dynamics and trophic behaviour to avoid predators [8], [9], [10], albeit with significant costs for individual and population viability in some cases [11], [12], [13]. Prey species also exhibit significant physiological and developmental adaptation to compensate for constraints imposed by predators [13], and can reduce predation risk by shifting habitat or food targets in order to avoid unsafe and/or predator-rich habitats [10].

On the other hand, predators can influence prey through consumptive and non-consumptive effects (NCEs) [4], [12], [14] such as, for example, reduced amount of time spent foraging [15], or reduced prey local population densities via changes in immigration and emigration rates [12], [16]. Empirical and modelling evidence indicates that NCEs can play a major role in regulating and structuring natural communities [9], [12], [17]. In addition, meta-analyses show that NCEs can have major effects on prey population dynamics [18] and can dominate trophic cascades through changes in prey ecological traits [19]. Nevertheless, these studies also highlight the need for research into the trait- versus density-mediated effects of predators on prey-resource interaction, as well as advances in the direct observation of the final outcomes of NCEs at community level.

Prey responses to predators have frequently been addressed in theoretical and field studies exploring movement between habitats characterized by different prey escape success rates and resource exploitation possibilities [20], [21]. In contrast, much less attention has been paid to the effects of predator-prey interaction on prey movement and behaviour within single aquatic habitats, such as cobble/gravel or vegetated habitats [22], [23], or heaps of leaf litter accumulated on the bottom of rivers. In lotic systems, reduced movement has been shown to represent a behavioural response to predation risk [22], [24]. On the other hand, prey mobility, abundance and diversity have been shown to influence prey selection and attack rate by predators [25]. The overall impact of predator-prey interaction on population dynamics is species-specific and influenced by resource availability, habitat complexity and environmental conditions [26], [27], [28]. However, field studies of predator-prey interaction in lotic ecosystems showed high variability in the magnitude and direction of predator effects on prey abundance and prey-resource interaction [14]. Moreover, few field studies addressing the combined effect of high-density predators and resource quality on the trophic behaviour of coexisting detritivores and their nutrient uptake from resources have been conducted, despite the fundamental importance of detritus-based food webs and associated processes in freshwater ecosystems [5], [29]. Phosphorus in particular is a major factor limiting the productivity of freshwater ecosystems [30], [31], and the supply and transfer of this element depends heavily on the transfer of matter along detritus-based food chains [32].

To determine the role of predators in (i) the movement of coexisting freshwater detritivore species between different resource patches and (ii) Phosphorus circulation within a three-trophic-level system, we carried out an *in situ* manipulative experiment in the River Kelvin (Scotland). In enclosures/exclosures for predators and prey we explored the effects of two invertebrate predators, *Dendrocoelum lacteum* (Müller) (Platyhelminthes, Tricladida) and *Erpobdella octoculata* (L.) (Anellida, Hirudinea), on the movement and trophic niches of two coexisting prey species, the isopod *Asellus aquaticus* (L.) and the pulmonate snail *Lymnaea peregra* (Müller), feeding on five resource patches made up of leaf litter conditioned with different fungus strains [33]. The consequent effect on phosphorus transfer along the food chains was measured. We used ^32^P to label one of the five fungus-inoculated leaf-bags in each leaf sack to assess the proportion of detritivores using it as food and to quantify fungus-specific consumption by detritivores, predicting reduced prey movement and phosphorus uptake on resource patches in the presence of predators.

## Materials and Methods

### Ethics Statement

The experiment was carried out on the River Kelvin (55°58′05″ N; 4°02′15″W) (Glasgow, Scotland) during late Summer 1983. Approval of the experimental design (and of the total quantity of ^32^P used) was sought and obtained at the time from the Radiation Protection Service of the University of Glasgow. Current regulations would require permission from the Scottish Environmental Protection Agency (SEPA), but informal consultation with Glasgow University Radiation Protection Service indicates that the levels described fall within acceptable limits for discharge and that the project would likely be approved by SEPA if submitted today. No specific permits other than that owned by the Department of Zoology at the time of the research were required for the study described in the present paper. The tract of the river is not privately-owned or protected and the present study did not involve endangered or protected species. Only small invertebrates and dead leaves were studied, and no residuals of ^32^P were found in vegetation, detritus, sediments and animals outside the enclosures at the end of field experiment.

### Study Site and Experimental Design

The River Kelvin is 34 km long, flowing from the East of Kilsyth (48 m a.s.l.) to the River Clyde (Glasgow, 6 m a.s.l.), where it reaches a maximum width of 20 m. The floodplain is intensively farmed and, to a lesser extent, industrialized. The river banks are characterized by a narrow band of trees dominated by alders (*Alnus glutinosa*), while wider patches of riparian vegetation are observed along the river course only occasionally. Our experiment was carried out on the central-western stretch of the river, which is free of artificial banks and characterized by natural alder leaf litter accumulation. Invertebrate species were collected at the same study site used for the experiment.

Two prey species were used: the isopod *Asellus aquaticus* and the pulmonate snail *Lymnaea peregra*, both of which are known to eat fungi on leaf litter. These species were the most abundant among macroinvertebrates colonizing leaf litter in the River Kelvin, with *A. aquaticus* naturally 2 to 3 times more abundant than *L. peregra*. The body length of *A. aquaticus*, which is more vagile than *L. peregra,* ranged between 8 and 15 mm from the base of antennas to the distal part of the telson, whereas the shell length of *L. peregra* ranged between 7 and 12 mm. Their predator species in the experiment were *Dendrocoelum lacteum* (Platyhelminthes, Tricladida) and *Erpobdella octoculata* (Anellida, Hirudinea), both common generalist invertebrate predators, easily collected from the surface of submerged stones in the river. The body length of *D. lacteum* and *E. octoculata* ranged between 17 and 24 mm and between 25 and 35 mm respectively. The two predators are known to have different feeding strategies: *D. lacteum* has a more sit-and-wait behaviour with respect to *E. octoculata*, which pursues prey more actively [34], [35]. Both species are known to feed on *A. aquaticus* and, to a lesser extent, on *L. peregra* [36], [37], [38], [39], [40], relying on their chemosensory system, more developed in triclads than in leeches, to detect prey [34]. Their prey selection depends on prey abundance and encounter frequency [37], [39].

**Figure 1:**
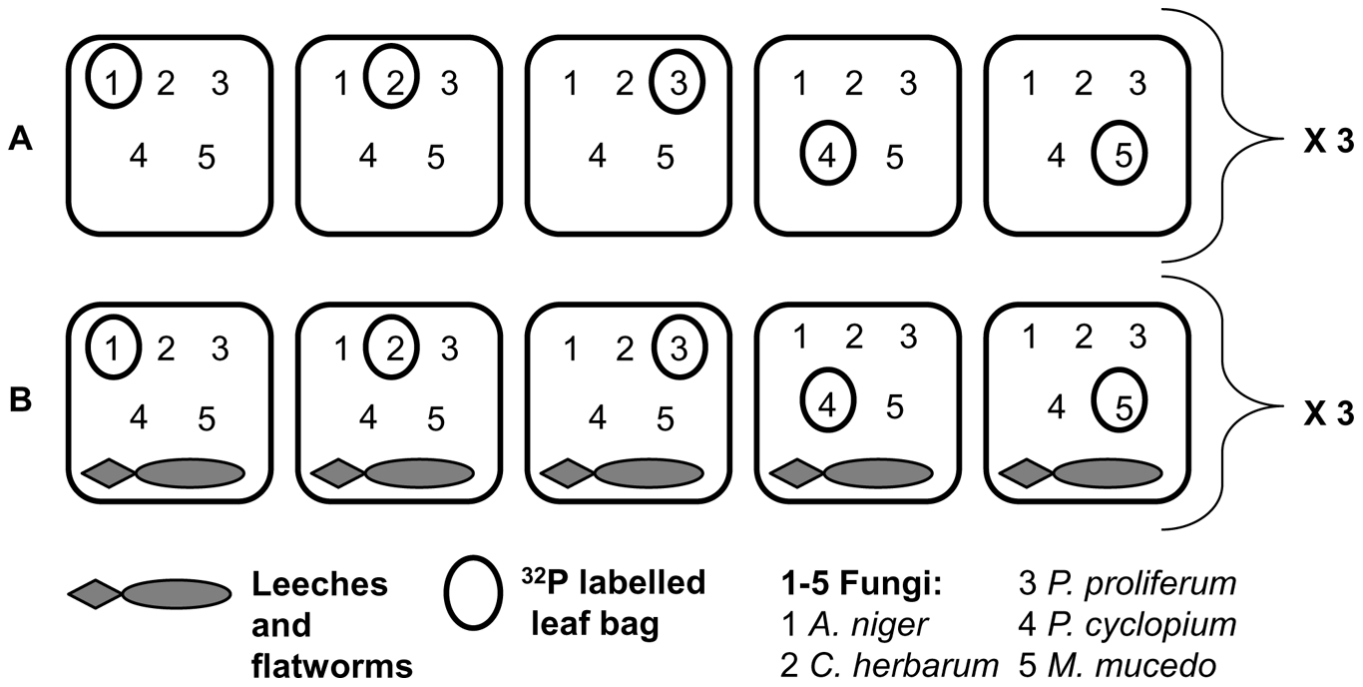
Experimental design. Two experimental sets (A = detritivores only and B = detritivores with predators) in triplicate, six enclosure/exclosure cages in total. Each replicate set included 5 leaf sacks (black squares), each containing 5 leaf bags individually inoculated with different pure fungus strains (1–5). In each leaf sack only one leaf bag was labelled with 32P (circled number).

The experiment was performed in six 1-mm mesh enclosure/exclosure cages (70×150×250 cm) compartmented in 5 sections. Each section was filled with about 1.2 wet-kg of alder leaf detritus, which was collected from the study site, carefully washed to remove animals while preserving microbial colonization, and then weighed with a portable balance (precision ±0.5 g). The 1-mm mesh size of the cages prevented predator and prey escape from and entry to the cages, while allowing water exchange and access only to other species with a body size under 1 mm. Five leaf sacks (30×60×80 cm, 1-cm mesh size), each containing 0.6 wet-kg of alder leaf detritus, mimicking natural heaps of detritus, were added to each cage. They were separated by a fine mesh (1 mm) to prevent animals moving from one leaf sack to the others (Fig. 1), whereas the 1-cm mesh size allowed the invertebrates to move in and out of each sack.

About 1200 *A. aquaticus* individuals and 450 *L. peregra* individuals were placed in each leaf sack. Then five alder leaf bags (4.5 g dry weight, mesh size 1 cm, hereafter also referred to as “resource patches”), containing freshly fallen leaves collected from the river, each of which was incubated with one of five fungal strains in order to differentiate the detritivore resources (see next section), were added to each leaf sack. The five fungal strains (*Aspergillus niger*, *Cladosporium herbarum*, *Pytium proliferum*, *Penicillum cyclopium*, *Mucor mucedo*) were isolated from alder leaf litter collected from the study site.

To manipulate the presence/absence of invertebrate predators, *D. lacteum* and *E. octoculata* were added to half of the leaf sacks by introducing four cobbles (12-cm diameter) collected from the study site, each colonized by 30–35 specimens in a ratio of about one to one. Four cobbles without predators were added to each of the remaining leaf sacks. The number of predator and prey specimens used in the study was representative of the natural abundance of the species in the study area in that period (expressed as mean number of individuals per cobble and per gram of leaves respectively; L. Rossi, *personal observation*). The six experimental sets corresponding to 3 replicated cages for each of the 2 treatments (with and without predators), with 5 sacks per cage and 5 bags per sack, were left on the riverbed (80 cm depth) for 7 days (Fig. 1). To avoid the possible confounding effects of water flow on predator impact on both prey density and movement [41], we selected a slow-flowing stretch of the River Kelvin, making sure that the cages did not affect the flow regime. Upon leaf retrieval, colonizing animals were carefully sorted and transported to the laboratory.

### Leaf Litter Conditioning and Radio-labelling before the Experiment

Leaf bags (five per leaf sack) were conditioned by incubating each of them with one of five different fungus strains in accordance with [42]. Thus, 150 leaf bags, previously sterilized by autoclaving, were conditioned and gently stirred for 15 days at 15°C in 30 Erlenmeyer flasks (six flasks per fungus), each containing 1.5 l of distilled water enriched with 0.33 g 1^−1^ of CaC1_2_ and 0.12 g 1^−1^ of Na_2_CO_3_, and a suspension of fungal spores and hyphae. Autoclaving the litter leads to changes in chemical composition that mimics those due to leaching and it is therefore generally chosen as the best sterilization method [43]. While chemical changes influence fungal growth on litter, the palatability of litter for detritivores increases with the declining concentrations of tannins and polyphenols due to leaching and varies with the microbial conditioning. In order to obtain at the end of the incubation period an average fungal mass per gram of leaf litter in the leaf bags comparable to that of the naturally leached detritus outside the bags, as determined in preliminary dilution tests by counting the relative hyphae and spores under a microscope, a 10-ml suspension obtained from stirring two 9-mm disks from a pure fungal culture in 1 l of enriched water was added to each flask. To one of the six flasks for each fungus ^32^P as H_3_PO_4_ was added to a final concentration of 1.88 µCi/ml. Thus only one of the five leaf bags (and of the five fungus strains) in each leaf sack was labelled with ^32^P and there were three replicate leaf sacks for each labelled fungus and each experimental condition (predation/no predation) (Fig. 1).

### Radioactivity Measurements after the Experiment

Once transported to the laboratory, the level of ^32^P in each sample animal was measured by Geiger-Müller counter (CPM) to determine the percentage of prey animals that were labelled with ^32^P in each leaf sack. All individuals were then washed, oven-dried at 60°C for 3 days and weighed.

In accordance with [42], animals were set individually in glass vials and dissolved in 1 ml NCS-H tissue solubilizer (Amersham) in the dark for 24 h at 50°C in a water bath. The ^32^P level in each leaf bag was measured before exposure to animals by randomly cutting five leaf disks of 8 mm diameter and dissolving them in 1 ml NCS-H for 48 h in the dark. Radioactivity was determined by liquid scintillation counting in a Tricarb 4000 B counter after addition of glacial acetic acid and 10 ml of toluene scintillator to all vials. Counting efficiency was determined with reference to an external standard, and the results were corrected against blank samples. A further correction was made for radiotracer decay. The results were expressed as disintegration/min (1 DPM = 4.55×10^−13^ Curie) and, once converted, as ‘Activity Density’ (AD = µCi/g of sample dry-weight) [44]. The ‘Trophic Transfer index’ (TTI = consumer AD/resource AD) was then calculated in order to determine the amount of ^32^P transferred by a unit weight of consumer from a unit weight of each resource patch [modified from 42].

### Impact of Predators on Prey

For each of the two detritivore species, predator impact on (i) prey abundance (PI_Nc_), (ii) prey movement (PI_%L_), and (iii) prey Activity Density (PI_AD_) associated with each of the five resource patches was computed by considering (i) the mean number of specimens on the labelled leaf bags (Nc), (ii) the mean percentage of specimens labelled with ^32^P (%L) and (iii) the mean activity density (AD) of labelled specimens in the corresponding leaf sacks. Thus the mean Nc on each resource patch was calculated as the average value across the three replicate leaf bags for each of the five labelled fungus strains for each treatment (i.e. with or without predators), whereas the mean %L and AD were calculated as the average values across each three-replicate leaf sacks for each labelled fungus strain and each treatment. This made it possible to detect prey that ate on the labelled resource patch during the 7 days of the experiment but may not have been on the labelled leaf bag on retrieval.

In accordance with [40], PI_Nc_, PI_%L_ and PI_AD_ on each resource patch were determined as:
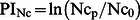
(1)

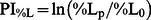
(2)

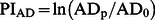
(3)where the subscripts P and 0 referred to predator presence and absence respectively. Such measures of predator impact avoid the possible confounding effects of different species-specific prey densities [45].

## Results

**Figure 2:**
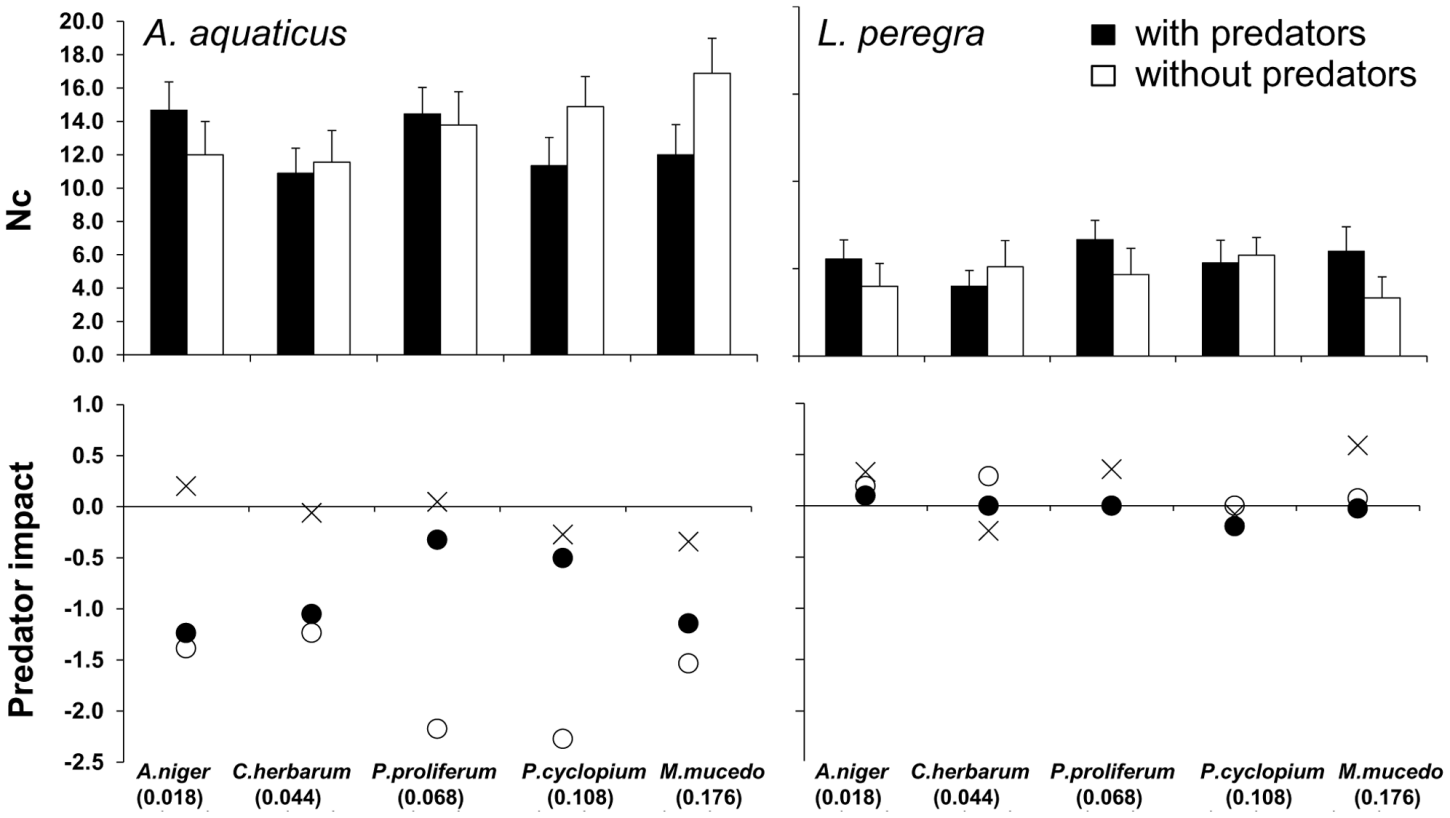
Predator impact on prey traits. Above: number of A. aquaticus and L. peregra specimens per gram of leaf litter (Nc) in leaf bags conditioned with one of five fungal strains, with predators (black bars) and without predators (white bars); below: predator impact on prey density (crosses), movement (•, based on percentage of prey labelled with 32P) and Activity Density (○). Numbers in parentheses on abscissa indicate Activity Density (µCi/g) of leaf bags conditioned with different fungus strains.

Each fungus strain took up the radiophosphorus from the incubation media at a different rate, reflecting its rate of colonisation and development (Fig. 2). The Activity Density (AD) of inoculated leaf bags ranged from 0.176 µCi/g for *Mucor mucedo* to 0.018 µCi/g for *Aspergillus niger*. In each leaf sack the non-labelled leaf bags remained completely unlabelled at the end of the experiment. The presence of predators had negligible effects on the mean species abundance of the detritivorous species on the resource patches (paired t-test t<1.2, *p* value >0.25 for both species, Table 1) with a mean impact of PI_Nc_ = 0.09±0.08 S.E. for *A. aquaticus* and 0.19±0.15 S.E. for *L. peregra* (Fig. 2). In the absence of predators, the leaf bags where fungal colonization was highest (i.e. with the highest fungal AD) were the most heavily colonized by detritivores and were associated with the highest ^32^P uptake; specifically, the abundance of *A. aquaticus* in the leaf bags was directly correlated with the AD of fungi (R = 0.97, *p* value <0.01) and its own AD (R = 0.91, *p* value <0.01).

**Table 1:** Effect of predators on *Asellus aquaticus* and *Lymnaea peregra*.

		Nc	%L	AD	TTI
*A. aquaticus*	P	12.7±0.8	39±9.1	3.8±0.6	75.4±29.3
	NP	13.8±1.0	80.8±6.7	21.4±3.5	360.6±97.3
*L. peregra*	P	5.5±0.4	46.0±15.6	2.1±0.7	54.3±22.9
	NP	4.6±0.4	45.8±14.8	1.9±0.7	44.5±18.3
Predators		3.2±0.3	51.4±13.6	0.3±0.2	0.1±0.03

P and NP refer to leaf bags with and without predators, respectively. Nc: number of specimens colonising leaf bags per gram of leaf litter; %L: percentage of total prey specimens in each leaf sack labelled with ^32^P; AD: Activity Density (µCi/g); TTI: Trophic Transfer Index. Means ± S.E. are reported.

**Figure 3:**
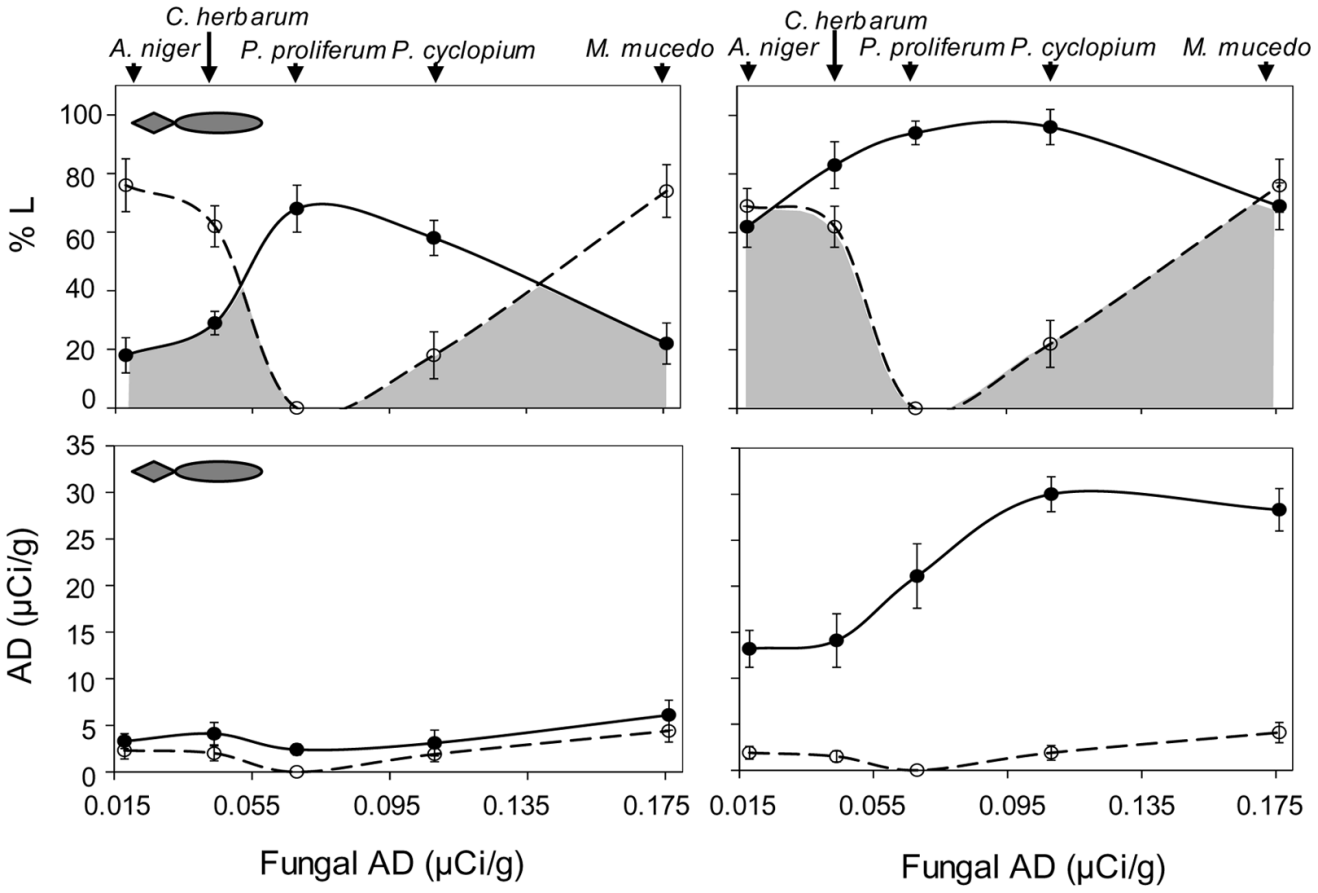
Effects of predators on prey movement and P uptake. Percentage of prey labelled with 32P (%L) on each resource patch (above), and 32P uptake from resource patches (AD of prey) (below) for Asellus aquaticus (solid line) and Lymnaea peregra (dashed line). Fungi are ranked by their AD, increasing from A. niger (AD = 0.018 µCi/g) to M. mucedo (AD = 0.176 µCi/g). Grey areas indicate trophic niche (as preference for specific resources) overlap between A. aquaticus and L. peregra.

There were considerable differences between the five labelled fungi in terms of the %L of detritivores exposed to them (Fig. 3). The percentages of labelled *A. aquaticus* and *L. peregra* were negatively correlated with each other, both in the absence (R = −0.88, *p* value <0.05) and presence of predators (R = −0.99, *p* value <0.001). Specifically, labelled specimens accounted for higher percentages at intermediate fungal AD for *A. aquaticus* and at low and high fungal AD for *L. peregra*. The %L of both species was not related to the number of specimens found in the leaf bags at the end of the experiment (Nc), in either the absence or presence of predators (*p* value always >0.05).

The presence of predators caused a significant reduction in the %L of *A. aquaticus* relative to all five resource patches (paired t-test t = 8.9, *p* value <0.001), but had no significant effect on *L. peregra* (paired t-test t = 0.1, *p* value = 0.9) (Table 1 and Fig. 3). The mean predator impact on the isopods was PI_%L_ = −0.93±0.1 S.E., which is 11 times higher than PI_Nc_ (paired t-test, *p* value <0.01), whereas the predator impact on the snails was PI_%L = _0.08±0.18 S.E. (Fig. 2). The greatest decline in the %L of *A. aquaticus* was seen in the leaf bags with the highest %L of *L. peregra*, meaning that the trophic niche overlap between the two detritivorous species was strongly reduced in the presence of predators (Fig. 3). At the end of the experiment 51.4±3.6% of predators were found to be labelled (Table 1).

The AD of *A. aquaticus* was always higher than that of *L. peregra* (Fig. 3). The presence of predators caused a significant reduction in the AD of the former (paired t-test t = 5.3, *p* value <0.005, Fig. 2 and Fig. 3), with a mean predator impact of PI_AD_ = −1.72±0.2 S.E., corresponding to a decrease in AD of about 42%, but had no significant effect on the latter (paired t-test t = 2.2, *p* value = 0.8; PI_AD = _0.11±0.05 S.E.). For values of Nc, AD, %L and predator impact on prey please see also supplemental materials (Tables S1 and S2).

**Figure 4:**
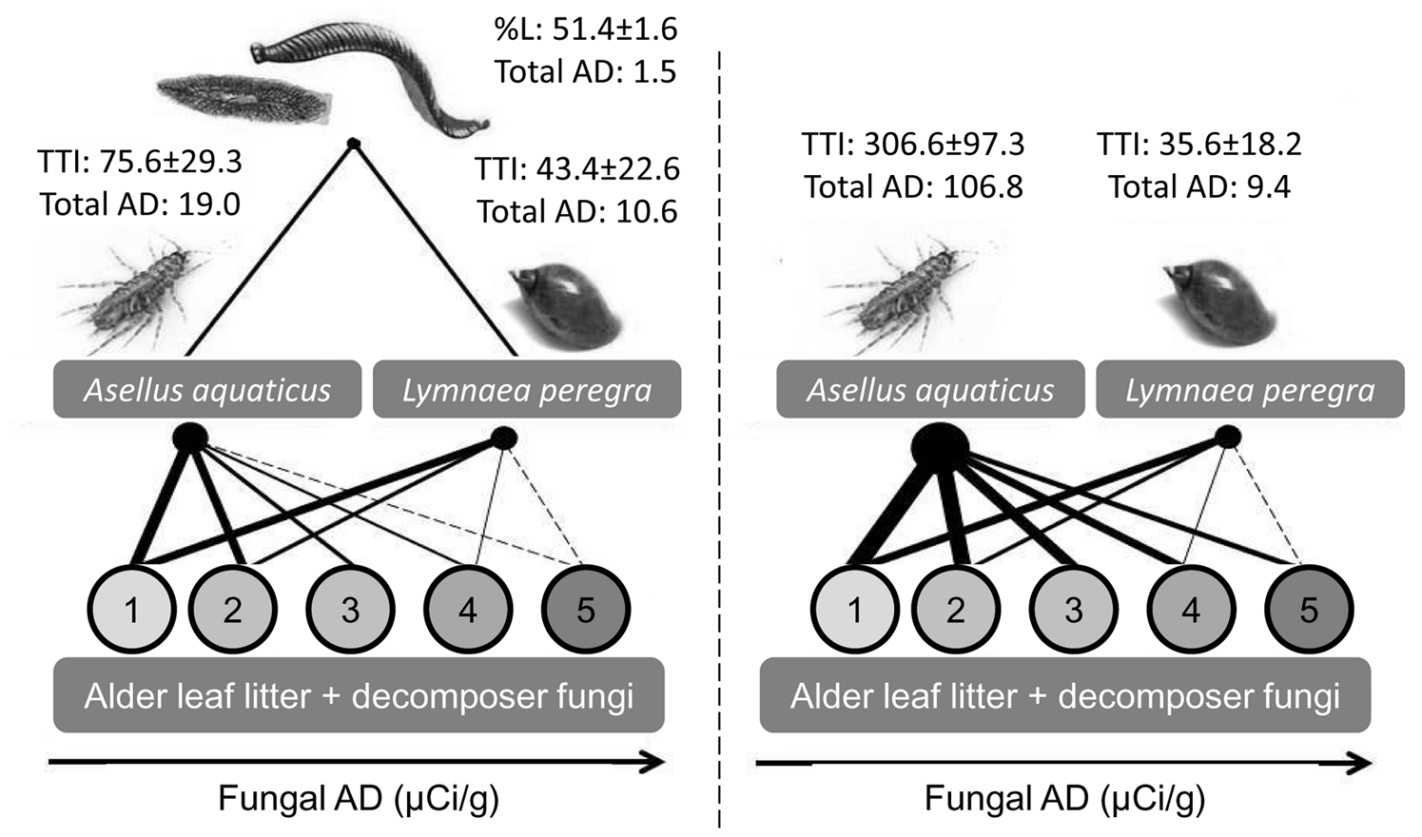
32P pathways in river Kelvin food web with (left panel) and without (right panel) predators. Basal resources, i.e. fungi colonizing leaf bags: 1 = A. niger, 2 = C. herbarum, 3 = P. proliferum, 4 = P. cyclopium, 5 = M. mucedo. Fungi are ranked according to Activity Density (AD), increasing from 1 (AD = 0.018 µCi/g) to 5 (AD = 0.176 µCi/g). Total AD = cumulative prey Activity Density for all five basal resources. TTI = mean prey Trophic Transfer Index from five basal resources (±S.E.). Black bar thickness is proportional to TTI values. %L = percentage of labelled predators.

**Figure 5:**
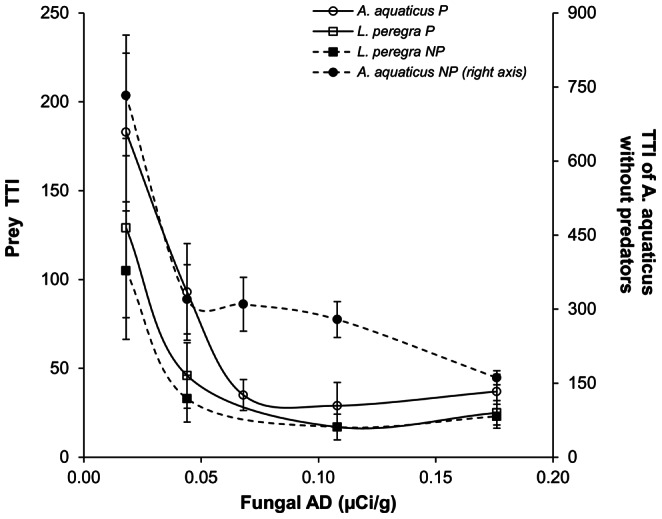
AD of fungi and TTI of detritivores. Relationship between fungal AD (µCi/g) and Trophic Transfer Index of Asellus aquaticus and Lymnaea peregra in presence (P) and absence (NP) of predators. Note that for A. aquaticus in absence of predators TTI values are reported on right axis.

In the presence of predators, the consumption of the five resource patches by *A. aquaticus* was more homogeneous than in the absence of predators (Fig. 3); together with the declining P uptake, this altered the quantity and pattern of phosphorus transfer through the system (Fig. 4). The presence of predators lowered the mean Trophic Transfer index (TTI) of *A. aquaticus* (paired t-test t = 4.1, *p* value = 0.01; Table 1 and Fig. 5) from 360.6±97 to 75.4±29 (80±4% reduction), whereas no effect of predation on the TTI of *L. peregra* was observed. The TTI of both species was negatively correlated with the AD of fungi, in both the presence and absence of predators (*p* value always <0.05, Fig. 5).

## Discussion

The results show the significant effect of predator presence on detritivorous prey movement and trophic behaviour and the combined effect of predators and resource quality on phosphorus uptake by prey from the ^32^P-labelled leaf detritus. The observation of ^32^P-labelled predators indicates that predators fed on prey during the 7 days of the experiment. Nevertheless, given the time-scale considered, the low density and feeding rates of predators and the initially high number of prey, predators had no statistically significant effects on the number of prey specimens. In contrast, the presence of predators reduced the proportion of labelled *A. aquaticus* specimens. Since five inoculated leaf bags were present in each leaf sack, but only one was labelled with ^32^P, the reduction of labelled animals is to be ascribed to the generally lower mobility of prey from one trophic patch to another when *D. lacteum* and *E. octoculata* were present [12].

The fact that the non-labelled leaf bags in each leaf sack were still unlabelled at the end of the experiment proves that ^32^P was well fixed in the leaf litter tissue and was not distributed throughout the leaf bags by physical factors (e.g. water movement). This implies that the prey were able to assimilate ^32^P only by colonizing and feeding on labelled resource patches. Given this, the variation in the proportion of labelled detritivores with respect to the different labelled fungus strains indicates differences in trophic preferences. The use of ^32^P as a tracer of detritivore nutrient uptake from different resources proved to be a reliable tool for quantifying detritivore movement among detritus patches and detritivore-fungi interaction during the field experiment. Animals were exposed to labelled food sources at very low concentrations, normally used in these kinds of experiments (since [44]), which are very safe for the environment and irrelevant for animal behaviour, feeding and fitness as observed in other studies [42], [46] and in laboratory. Because of the rapidity with which ^32^P is assimilated and transferred along the food chain, P-radiolabelling is very useful to perform short-term experiments necessary to prevent microbial contamination and autogenic changes in the basal resource patches. This goal could be hardly achieved with environmentally safer labelling techniques such as addition of N stable isotope, which needs much longer time to be detected due to assimilation, isotopic fractionation and tissue turn-over [47], [48].

In the absence of predators, the density of isopods was correlated to their AD, which in turn reflected the AD, and thus the growth rate, of fungi on alder leaf litter [49], [50], indicating a bottom-up (i.e. resource quality) control on prey abundance and phosphorus uptake. The relationship between fungal AD and the number of isopods on the resource patches was not significant in the presence of predators, indicating prevalently top-down control over prey movement and distribution [51]: the isopods were less mobile, and thus had less opportunities to exploit the most richly colonised leaves. Anti-predator responses among aquatic animals have been attributed to chemical cues signalling predator presence or injured prey, e.g. by detecting predator mucus, exudation or damaged conspecific fluids, as observed also in *Asellus* and *Lymnaea* [52], [53], [54], [55], coupled in some cases with visual or hydrodynamic cues [56], [57]. In addition, behavioural changes in response to warning cues have been demonstrated to represent an advantage for prey [58], [59], as they entail reduced activity in the presence of predators and hence decreased predation risk [60], [61].

Predators are expected to strike preferentially at abundant and/or vagile prey [3], [25], [62], [63]. Indeed, in our study predators induced decreased movement and resource consumption by *A. aquaticus*, which was the most abundant and vagile prey. No significant direct effects of predators on *L. peregra* were observed. In contrast, resource selection and consumption by *L. peregra* seem to have been constrained by the presence of *A. aquaticus*, as confirmed by the inverse relationship between the proportion of labelled specimens of *L. peregra* and *A. aquaticus* on the five resource patches, in both the absence and presence of predators. In the presence of predators, the trophic overlap between the two detritivore species decreased as a result of decreased movement and consumption of the five resource patches by *A. aquaticus*. Thus, in our experiment the presence of predators appears to have shielded *L. peregra* from competition with *A. aquaticus* by constraining the trophic behaviour but not the density of the latter. [64] demonstrated that top-down (predator) and bottom-up (resource) forces can symmetrically influence prey coexistence depending on species fitness and niche overlap. Consistent with theoretical evidence, the results of this study suggest that combined top-down and bottom-up controls underlie the coexistence of *A. aquaticus* and *L. peregra* in the River Kelvin as well as the flux of nutrients along the food chains in the three-trophic-level detritus-based system under study. Decreased mobility of isopods not only resulted in a lower proportion of individuals feeding on multiple resource patches but also in lower nutrient uptake, which was ascribable to the reduced feeding activity on each colonised resource patch in the presence of predators. Indeed, predator presence has been shown to reduce the time spent by prey feeding on resources due to increased time spent on avoiding predators and remaining inconspicuous even when colonizing a suitable resource patch [13], [17], [65].

While the AD of prey increased with resource quality, their TTI was highest on those resource patches where resource quality was lowest (indicated by lowest fungal AD). It has been suggested that lower resource quality leads to faster consumption of basal resources by consumer species, as a physiological adaptation to compensate [13], [66]. In our experiment, faster consumption by detritivores on low-quality resource patches did not enable them to achieve the same phosphorus uptake as gained when feeding on high-quality resource patches. By constraining movement and resource consumption by detritivores, the presence of predators exacerbates the negative effect of scarce resource quality on nutrient uptake and P transfer from detritus to upper trophic level.

Interestingly, the three-trophic-level system that we addressed in this study is representative of a broad geographical scale [39], [67], [68], [69]. Specifically, [39] showed that the distribution of *A. aquaticus* in 151 Swedish lakes was driven by the amount of leaf litter input and that, in turn, *A. aquaticus* abundance influenced the presence of *D. lacteum*. Given the site-scale characterising our experiment, further experiments considering the potential variability of the observed top-down and bottom-up controls across different spatial scales [70] will allow to understand if the observed effect of predators and resources quality on P recycling by prey is consistent at broader geographical scales. In addition, the trophic interactions in such detritus-based systems have been shown to be sensitive to water pollution and acidification during laboratory experiments [26], [71], [72]. This posits the comprehension of top-down and bottom-up combined effect on P recycling in lotic systems as a key step to understand how perturbation of species interactions could rebound at ecosystem level [30], [73], [74], [75]. In this perspective, the use of ^32^P in detritus-based systems makes it possible: (i) to investigate the effect of predators on prey movement, trophic behaviour and coexistence in a habitat only seemingly homogeneous, and (ii) to quantify nutrient transfer along food chains from single basal resources by detritivorous species. This could provide useful results for the understanding of the effects of top-down and bottom-up control on the community structure and the associated ecological processes at multiple spatial scales and under different environmental conditions.

## Supporting Information

Table S1 Mean values (±S.E.) of (i) number of specimens per gram of leaf litter (Nc), (ii) percentage of labelled specimens in each leaf sack (%L), and (iii) Activity Density (AD, in µCi/g) for *A. aquaticus* and *L. peregra* both when in the presence and absence of predators.
(DOCX)

Table S2 Mean values (±S.E.) of (i) number of specimens per gram of leaf litter (Nc), (ii) percentage of labelled specimens in each leaf sack (%L), and (iii) Activity Density (AD, in µCi/g) for predators. Predator impact on (i) prey density on resource patches (PI_Nc_), (ii) percentage of labelled prey (PI_%L_), and (iii) Activity density of prey (PI_AD_) are also reported.
(DOCX)
