# Supplementary material for: Predators and Resources Influence Phosphorus Transfer along an Invertebrate Food Web through Changes in Prey Behaviour
Source: PLoS One. 2013 Jun 4;8(6):e65186. doi: 10.1371/journal.pone.0065186 (PMC3672138; doi:10.1371/journal.pone.0065186)
Supplement: Table S1 — Mean values (±S.E.) of (i) number of specimens per gram of leaf litter (Nc), (ii) percentage of labelled specimens in each leaf sack (%L), and (iii) Activity Density (AD, in µCi/g) for A. aquaticus and L. peregra both when in the presence and absence of predators. (DOCX) [file pone.0065186.s001.docx]

Table_S1: Mean values (±S.E.) of (i) number of specimens per gram of leaf litter (Nc), (ii) percentage of labelled specimens in each leaf sack (%L), and (iii) Activity Density (AD, in µCi/g) for *A. aquaticus* and *L. peregra* both when in the presence and absence of predators.

|  |  | | |  |  | | |
| --- | --- | --- | --- | --- | --- | --- | --- |
| **Cages with predators** | *A. aquaticus* | | |  | *L. peregra* | | |
| Fungus strain | Nc | %L | AD |  | Nc | %L | AD |
| *A. niger* | 14.7±1.7 | 18±6 | 3.3±0.8 |  | 5.6±1.1 | 76±9 | 2.3±0.9 |
| *C. herbarum* | 10.9±1.5 | 29±4 | 4.1±1.2 |  | 4.0±0.9 | 62±7 | 2.0±0.8 |
| *P. proliferum* | 14.4±1.6 | 68±8 | 2.4±0.6 |  | 6.7±1.1 | 0±0 | 0.0±0 |
| *P. cyclopium* | 11.3±1.7 | 58±6 | 3.1±1.4 |  | 5.3±1.3 | 18±8 | 1.9±0.8 |
| *M. mucedo* | 12.0±1.8 | 22±5 | 6.1±1.6 |  | 6.0±1.4 | 74±9 | 4.4±1.2 |
| **Cages without predators** | *A. aquaticus* | | |  | *L. peregra* | | |
| Fungus strain | Nc | %L | AD |  | Nc | %L | AD |
| *A. niger* | 12.0±2.0 | 62±7 | 13.2±2.2 |  | 4.0±1.3 | 69±6 | 1.9±0.7 |
| *C. herbarum* | 11.6±1.9 | 83±8 | 14.1±3.1 |  | 5.1±1.5 | 62±7 | 1.5±0.6 |
| *P. proliferum* | 13.8±2.0 | 94±4 | 21.1±3.7 |  | 4.7±1.5 | 0±0 | 0.0±0.0 |
| *P. cyclopium* | 14.9±1.8 | 96±6 | 30.0±3.9 |  | 5.8±1.0 | 22±8 | 1.9±0.8 |
| *M. mucedo* | 16.9±2.1 | 69±8 | 28.3±2.5 |  | 3.3±1.2 | 76±9 | 4.1±1.2 |
|  | |  |  |  |  |  |  |
|  | | | |  |  |  |  |
|  | |  |  |  |  |  |  |

|  | |  | | |  |  | | |  | |  | |  | |  |  |  |
| --- | --- | --- | --- | --- | --- | --- | --- | --- | --- | --- | --- | --- | --- | --- | --- | --- | --- |
|  |  | | |  |  |  | |  | |  |  |  |  |  |  |  |  |
|  |  | |  | |  |  |  | |  | | |  | |  |  |  |  |
|  | |  | | |  |  | | |  | |  | |  | |  |  |  |
