# Supplementary material for: Predators and Resources Influence Phosphorus Transfer along an Invertebrate Food Web through Changes in Prey Behaviour
Source: PLoS One. 2013 Jun 4;8(6):e65186. doi: 10.1371/journal.pone.0065186 (PMC3672138; doi:10.1371/journal.pone.0065186)
Supplement: Table S2 — Mean values (±S.E.) of (i) number of specimens per gram of leaf litter (Nc), (ii) percentage of labelled specimens in each leaf sack (%L), and (iii) Activity Density (AD, in µCi/g) for predators. Predator impact on (i) prey density on resource patches (PINc), (ii) percentage of labelled prey (PI%L), and (iii) Activity density of prey (PIAD) are also reported. (DOCX) [file pone.0065186.s002.docx]

Table_S2: : Mean values (±S.E.) of (i) number of specimens per gram of leaf litter (Nc), (ii) percentage of labelled specimens in each leaf sack (%L), and (iii) Activity Density (AD, in µCi/g) for predators. Predator impact on (i) prey density on resource patches (PI_Nc_), (ii) percentage of labelled prey (PI_%L_), and (iii) Activity density of prey (PI_AD_) are also reported.

|  |  |  |  |  |  |  |  |  |  |  |  |
| --- | --- | --- | --- | --- | --- | --- | --- | --- | --- | --- | --- |
|  | Predators | | |  | *A. aquaticus* | | |  | *L. peregra* | | |
| Fungus strain | Nc | %L | AD |  | PI_Nc_ | PI_%L_ | PI_AD_ |  | PI_Nc_ | PI_%L_ | PI_AD_ |
| *A. niger* | 3.3±0.9 | 16±4 | 0.1±0.05 |  | -0.2 | -1.2 | -1.4 |  | -0.3 | 0.1 | 0.2 |
| *C. herbarum* | 3.1±0.8 | 72±5 | 0.2±0.04 |  | 0.1 | -1.1 | -1.2 |  | 0.2 | 0.0 | 0.3 |
| *P. proliferum* | 3.6±1.3 | 23±4 | 0.1±0.03 |  | 0.0 | -0.3 | -2.2 |  | -0.4 | 0.0 | 0.0 |
| *P. cyclopium* | 3.8±1.1 | 61±6 | 0.3±0.13 |  | 0.3 | -0.5 | -2.3 |  | 0.1 | -0.2 | 0.0 |
| *M. mucedo* | 2.2±0.9 | 85±3 | 1.0±0.20 |  | 0.3 | -1.1 | -1.5 |  | -0.6 | 0.0 | 0.1 |
